# Effects of biochar and manure amendments on soil microbial communities and crop yield in yellow cinnamon soil

**DOI:** 10.3389/fpls.2026.1884267

**Published:** 2026-07-07

**Authors:** Hui Li, Mengyuan Wang, Feikong Song, Jianing Zhang, Tengfei Guo, Hongen Liu, Mingyu Wu

**Affiliations:** 1 Hezhou University, Hezhou, Guangxi, China; 2 Resources and Environment College, Henan Agricultural University, Zhengzhou, China; 3 Institution of Plant Nutrition and Environmental Resources, Henan Academy of Agricultural Sciences, Zhengzhou, China

**Keywords:** biochar, manure, microbial community composition, microbial diversity, yellow cinnamon soil

## Abstract

**Introduction:**

Long-term chemical fertilization degrades soil properties and reduces microbial diversity, posing a significant threat to sustainable crop production. Yellow cinnamon soil is widespread across China, yet its weak structure and lower fertility substantially constrain crop productivity.

**Methods:**

To address this challenge, we conducted a long-term experiment on yellow cinnamon soil to evaluate the individual and synergistic effects of biochar and manure amendments on soil fertility, microbial communities and wheat yield. Six treatments were established, namely unfertilized control (CK), biochar alone (B), mineral fertilizer (NPK), biochar plus mineral fertilizer (NPKB), partial manure substitution for mineral N (NPKM), and combined biochar with partial manure substitution (NPKMB).

**Results:**

Our results showed that organic amendments markedly improved soil fertility, crop productivity, and microbial community structure. NPKM significantly elevated nutrient contents, alleviated acidification induced by mineral fertilizers, and reduced soil bulk density, and ultimately raised wheat and maize yield by 6.68% and 9.12%, respectively. NPKMB amplified these benefits and raised wheat and maize yield by 85.56% and 67.64% over the CK treatment. With respect to microbial responses, exclusively mineral fertilization lowered microbial diversity and altered community composition, whereas adding biochar and manure restored microbial richness and diversity to levels comparable to those in unfertilized soil. Notably, the NPKMB treatment enriched potentially beneficial biomarkers, including *Devosia, Porphyrobacter, Reyranella, Trechispora*, and *Pyrenochaetopsis*.

**Discussion:**

These taxa correlated positively with AK, AP, NH₄⁺-N, SOC, underscoring their potential roles in nutrient mobilization and plant growth promotion. Overall, NPKMB offers an effective sustainable strategy for managing yellow cinnamon soil. It improves soil physical and chemical quality, stimulates microbial activity, enriches beneficial taxa, and ultimately enhances wheat yield. These findings support optimized fertilization practices that enhance soil health and ecosystem function in yellow cinnamon soil regions.

## 1 Introduction

Agricultural management is essential to maintaining soil quality, stability, and sustainable crop production (Ţopa et al., 2025). However, prolonged chemical fertilization progressively degrades soil nutrients and physical structure, causing severe fertilizer leaching, SOM depletion, acidification, and compaction (Liang et al., 2019). Crop growth suffers as a result (Curtin and Trolove, 2013) while soil microbes face stronger environmental filtering that disrupts their metabolism (Han et al., 2022). Given that microorganisms drive biochemical processes and mediate soil functions, their community succession largely determines soil quality (Tang et al., 2022). Moreover, exclusively chemical fertilization disrupts soil nutrient balance, notably lowering the C: N ratio and accelerating acidification. Once soils become acidic and carbon-poor, carbon metabolism shifts from biological assimilation to respiratory loss (Duan et al., 2024), further depleting SOC and trapping soil ecology in a vicious cycle of degradation. Therefore, sustaining soil quality while ensuring productive agriculture remains a central research priority (Liu et al., 2021).

Organic fertilizers can improve soil quality, increase SOM, and raise the yields of staple crops such as rice, wheat, and corn (Liu et al., 2021; Li et al., 2022; Liu et al., 2022), primarily by enhancing soil nutrients, properties and microecology (Liu et al., 2021). Manure not only elevates SOC and enriches nutrients, but also improves soil porosity and structural stability, thereby stimulating microbial activity and accelerating nutrient cycling for better crop production (Lian et al., 2022; Liu et al., 2021). Beyond enhancing productive soils, manure application can also restore crop productivity and long-term sustainability to degraded soils (Liu et al., 2021). Shahid et al. (2017) further revealed that combining chemical fertilizers with manure optimizes both crop productivity and carbon storage. Moreover, partial replacement of inorganic inputs with organic alternatives changes soil physicochemical properties and enzyme activity, thus reducing or even reversing the damage from long-term exclusive chemical fertilization (Qaswar et al., 2020).

Biochar, produced mainly from agricultural waste by pyrolysis, is another promising amendment for improving soil quality and productivity (Dong et al., 2024). It enhances soil properties, nutrient availability, microbial activity, and crop yield (Akhil et al., 2021), largely by SOC and humic acid levels (Cooper et al., 2020). Biochar also stabilizes SOM, thereby improving water-holding capacity and nutrient retention (Yang et al., 2023). These carbon gains can shift microbial community structure (Dong et al., 2024), often enriching phyla such as *Proteobacteria*, *Actinobacteria*, and *Gemmatimonadota* (Azeem et al., 2021), all of which govern soil biogeochemical cycling. By altering soil characteristics and microbial habitats, biochar further influences microbial turnover and metabolism (Gorovtsov et al., 2020). Moreover, biochar-derived dissolved SOM can redirect soil metabolism toward mineralizing simple substances, which slows SOM decomposition and consequently alters soil quality (Azeem et al., 2023).

Yellow cinnamon soil is widespread across China, yet its weak structure and lower fertility substantially constrain crop productivity (Zhang, 2022). To date, few studies have focused on how yellow cinnamon soil responds to combined biochar and manure amendments, as most research has concentrated on paddy soils, fluvo-aquic soils, and lime concretion black soils (Cheng et al., 2023; Dong et al., 2024; Wu et al., 2025). The long-term effects of organic amendments (biochar and manure) on soil properties and microbial communities in this specific soil type remain poorly understood. In particular, it is still unclear how partial substitution of mineral nitrogen with manure, applied alone or in combination with biochar, affects: (i) long-term nutrient availability and soil physical structure; (ii) bacterial and fungal diversity and community composition; and (iii) the linkages between microbial biomarkers and wheat/maize yield under a wheat–maize rotation system.

## 2 Materials and methods

### 2.1 Field site

The experiment was conducted at Zhaohe Town, Nanyang City, Henan Province, China (33°08’ N, 112°58’ E). The site experiences a subtropical monsoon climate, with average annual precipitation of 800–1200 mm, mean annual temperature of 15 °C, and annual sunshine duration of 2010 h. The soil is yellow cinnamon soil derived from Q3 loess parent material (Gong et al., 2003). Local farmer follows a winter wheat-summer maize rotation typical of the region. Before the experiment began, the soil had the following baseline characteristics: pH 5.82, SOC 10.63 g·kg^-1^, available nitrogen (AN) 137.86 mg·kg^-1^, available phosphorus (AP) 41.68 mg·kg^-1^, available potassium (AK) 87.30 mg·kg^-1^, and bulk density 1.34 g·cm^-3^.

### 2.2 Experimental design and sampling

The experiment began in 2012 and included six treatments: (1) no fertilization (CK); (2) biochar only (B); (3) mineral fertilizer only (NPK); (4) mineral fertilizer plus biochar (same rates as NPK and B, respectively) (NPKB); (5) mineral fertilizer with 60% of N re-placed by chicken manure (NPKM); (6) mineral fertilizer plus chicken manure (same as NPKM) plus biochar (same as B) (NPKMB). The plot layout diagram, details on fertilizer rates, composition, and sources appear in **Supplementary Materials**—**Supplementary Figure 1** and **Text 1**. We arranged the treatments in a randomized complete block design, with each plot measuring 40 m² (5 m × 8 m) and three replicates per treatment, giving 18 plots total.

The experiment followed a winter wheat–summer maize rotation. Wheat (cv. Zhengmai 9023, Hefei Fengle Seed Industry Co., Ltd) and maize (cv. Nonghua 101, Beijing Jinsenonghua Seed Technology Co., Ltd) varieties remained unchanged throughout the study.

We assessed crop yield annually by sampling plants at physiological maturity. At wheat maturity, we collected soil and plant samples. Soil was taken from 0–20 cm depth in each plot, with five cores collected in an S-shaped pattern and mixed into one single composite sample after removing visible debris, stones, and roots. The soil was then gently sieved (2 mm mesh) and subdivided for DNA extraction and for physicochemical and microbiological analyses.

We measured soil pH, bulk density (BD), total nitrogen (TN), SOC, ammonium nitrogen (NH_4_^+^-N), nitrate nitrogen (NO_3_^--^N), AN, AP and AK. All analytical procedures are described in **Supplementary Materials Text 2**.

### 2.3 Soil DNA extraction and sequencing of bacterial and fungal communities

Genomic DNA was extracted from 0.50 g of mixed fresh soil using the FastDNA^®^ SPIN Kit (MP Biomedicals, Illkirch, France), with mechanical lysis in a FastPrep-24 instrument (MP Biomedicals, Irvine, CA). DNA purity and concentration were assessed using a NanoDrop ND-2000 spectrophotometer (Thermo Fisher Scientific, USA). The V3-V4 hypervariable region of the bacterial 16S rRNA gene was amplified using primers 338F (5′-ACTCCTACGGGAGGCAGCAG-3′) and 806R (5′-GGACTACHVGGGTWTCTAAT-3′), as described in the NCBI BioProject database (PRJNA877374). The fungal ITS region was amplified using primers ITS1F (5′-CTTGGTCATTTAGAGGAAGTAA-3′) and ITS2R (5′-GCTGCGTTCTTCATCGATGC-3′) (Mukherjee et al., 2014). PCR amplification was performed using the TransStart FastPfu DNA Polymerase according to the manufacturer’s recommendations. All amplicons were purified using a Gel Extraction Kit (Qiagen, Hilden, Germany) and pooled in equimolar amounts. Paired-end sequencing was performed on an Illumina MiSeq PE300 platform (Illumina, San Diego, CA, USA). Raw reads were assembled in FLASH (v1.2.11), merging paired reads that overlapped by at least 12 nucleotides with 100% identity. Low-quality bases and adapter sequences were trimmed in Trimmomatic (v0.38), and chimeric sequences were removed using UCHIME (v4.2) prior to downstream analysis. The raw sequence data have been deposited in the National Microbiology Data Center (NMDC) under BioProject accession number NMDC10020748.

### 2.4 Statistical analysis

Normally distributed data were analyzed by one-way ANOVA followed by Duncan’s multiple range test (*P* < 0.05) in SPSS 23.0 (IBM Corp., Armonk, NY, USA). We used Mothur (https://www.mothur.org/) and Qiime (http://qiime.org/) to plot species rarefaction curves and calculate bacterial and fungal α-diversity and β-diversity. Principal coordinate analysis (PCoA) and redundancy analysis (RDA) were performed using the ‘vegan’ package in R. Bray-Curtis-based PCoA revealed differences among fertilization treatments, while RDA evaluated how environmental factors shaped microbial communities. We performed linear discriminant analysis (LDA) effect size (Lefse) in R using the ‘ggtree’ package, setting the LDA threshold at 3, and considered only taxa with LDA scores > 3 as biomarkers. The Kruskal-Wallis test identified significant differences in microbial taxa (relative abundance > 0.1%) between treatments. Spearman correlation analysis was performed using the ‘corrplot’ package in R to explore the relationships among crop yield, microbial diversity, and environmental factors.

## 3 Results

### 3.1 Crop yield and soil chemical properties

**Figure 1:**
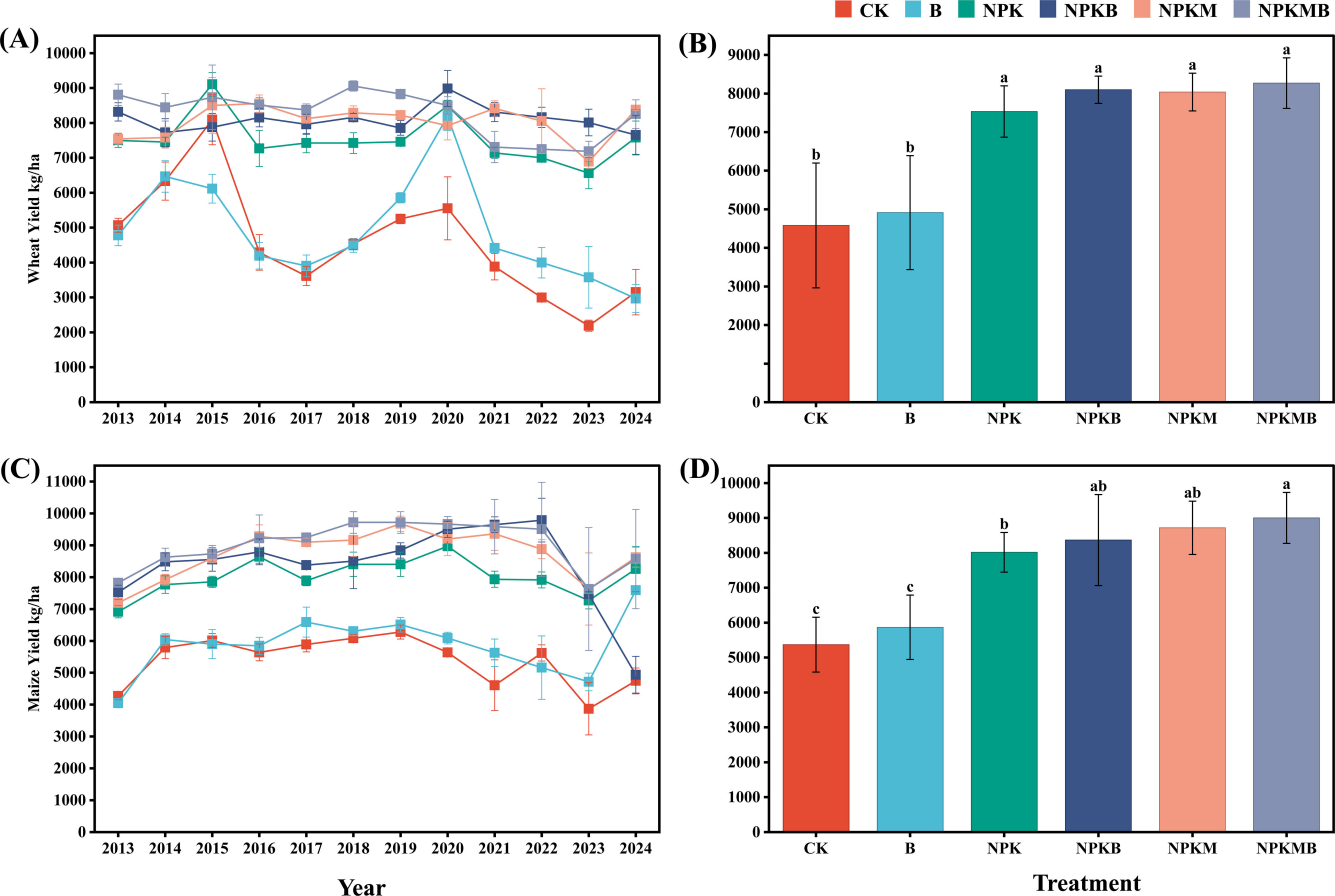
The changing trends of crop yields (**(A)** wheat; **(C)** maize) and the annual average yields [**(B)** wheat; **(D)** maize] under long-term different fertilization treatments. Error bars represent the standard error of the mean. Different lowercase letters indicate significant differences at *P* < 0.05.

Crop yields across all treatments exhibited distinct temporal patterns. Yields under the NPKB and NPKM treatments generally increased over time, whereas those under the NPKMB treatment remained relatively stable, with occasional declines in certain years. The annual yield growth rate in the CK and B treatments decreased over the study period, while NPKB, NPKM and NPKMB treatments exhibited significantly higher rates (*P* < 0.05; **Figures 1A, C**). The average annual yields of wheat and maize were highest under the NPKMB treatment, reaching 8499.70 kg·ha^−1^ and 9001.08 kg·ha^−1^, respectively, followed by the NPKM, NPKB, NPK, B, and CK (**Figure 1**). In yellow cinnamon soil, biochar amendment increased wheat and maize yields by 4.45-7.28% and 3.62-8.96%, respectively, compared to non-biochar controls. Among all treatments, NPKMB performed best, outperforming B and NPKB by 60.14% and 8.01% in wheat, and by 53.63% and 3.77% in maize, respectively. Among treatments without biochar, NPKM achieved the highest yields of 8038.61 kg·ha^−1^ for wheat and 8686.87 kg·ha^−1^ for maize, surpassing CK by 64.49% and 48.26% (*P* < 0.05), and NPK by 6.69% and 9.12%, respectively, demonstrating that combining organic and mineral fertilizers effectively boosts crop yield.

Soil chemical properties also responded distinctly to fertilization. The soil pH ranged from 5.15 to 5.50 (**Table A2**). Long-term mineral fertilization lowered pH significantly (*P* < 0.05), whereas biochar buffered this acidity, with NPKMB and NPKM showing the strongest effects (*P* < 0.05). CK had the highest bulk density (BD) at 1.49 g·cm^-3^, while all fertilized plots reduced this significantly (*P* < 0.05); NPKMB achieved the lowest BD at 1.35 g·cm^-3^. Apart from AP, biochar amended treatments generally carried higher nutrient levels than those without biochar, with SOC and AK rising markedly (**Table A2**). The AP in NPKB fell 2.81% below NPK, though this drop was not significant. AK followed the ranking of NPKMB > NPKM > NPKB > NPK > B > CK (*P* < 0.05), and fertilization raised AK above the CK group by 13.57%, 35.18%, 43.49%, 66.20%, and 97.23%, respectively. In addition, among biochar treatments, NPKMB contained the most nutrients, followed by NPKB and B, with significant differences in TN, SOC, NH_4_^+^-N, and AP (*P* < 0.05). The NO_3_^--^N level peaked under NPK, trailed by NPKMB and NPKB.

Spearman correlation analysis showed that wheat yield correlated significantly and positively with every environmental factor except BD and pH, wherein AK showed the strongest link, followed by NH_4_^+^-N, TN, AP and SOC (**Table 1**). Both pH and BD correlated negatively with yield and nutrient levels, with BD alone reaching significance.

**Table 1:** Spearman correlation coefficients between soil properties and wheat yield.

	Yield	TN	AN	NH_4_^+^-N	NO_3_^--^N	SOC	AP	AK	pH	BD
Yield	1									
TN	0.93***	1								
AN	0.74***	0.80***	1							
NH_4_^+^-N	0.97***	0.92***	0.73**	1						
NO_3_^--^N	0.54**	0.64**	0.78***	0.53*	1					
SOC	0.87***	0.90***	0.78***	0.87***	0.46**	1				
AP	0.92***	0.87***	0.73***	0.90***	0.64**	0.79***	1			
AK	0.98***	0.94***	0.79***	0.95***	0.59**	0.87***	0.95***	1		
pH	-0.20	-0.34	-0.22	-0.21	-0.58	0.12	-0.27	-0.23	1	
BD	-0.61*	-0.59**	-0.44**	-0.23*	-0.39**	-0.72***	-0.53**	-0.56**	-0.24	1

*P < 0.05, **P < 0.01, and ***P < 0.001.

### 3.2 Soil bacterial and fungal alpha-diversity

Long-term fertilization altered microbial alpha-diversity, spanning species diversity (Shannon and Simpson) and richness (ACE and Chao) (**Table 2**). Rarefaction curves approached saturation as sequencing depth increased (**Supplementary Figure 2**), indicating adequate coverage for characterizing the soil microbiome. For bacteria, the bacterial Shannon diversity was significantly lower under NPK than under CK (*P* < 0.05), whereas the ACE and Chao richness in all other treatments exceeded those than under NPK (*P* < 0.05). No significant differences were observed in the bacterial Simpson index among treatments (**Table 2**). For fungi, the Simpson diversity dropped significantly under each fertilization regime relative to CK (*P* < 0.05), while the Shannon diversity rose (*P* < 0.05), peaking under NPK. Fungal ACE and Chao indices did not differ significantly among treatments (**Table 2**). Overall, biochar addition supplied substrates for soil microorganisms, stimulating proliferation and increasing fungal richness and diversity, yet NPKM more effectively enhanced bacterial richness and diversity.

**Table 2:** Effects of long-term fertilization on soil microbial α diversity.

	Treatment	Shannon	Simpson	ACE	Chao
Bacteria	CK	6.036 ± 0.076a	0.009 ± 0.003a	2680.675 ± 92.361a	2691.839 ± 118.551a
	B	5.952 ± 0.098ab	0.013 ± 0.002a	2643.093 ± 46.160a	2601.830 ± 102.789a
	NPK	5.606 ± 0.222b	0.016 ± 0.008a	2087.489 ± 170.584b	2065.806 ± 187.752b
	NPKB	5.872 ± 0.039ab	0.014 ± 0.000a	2584.578 ± 98.611a	2576.491 ± 72.706a
	NPKM	5.811 ± 0.404ab	0.013 ± 0.009a	2528.241 ± 465.401a	2517.935 ± 478.848a
	NPKMB	5.967 ± 0.085ab	0.010 ± 0.003a	2581.303 ± 157.917a	2600.366 ± 205.880a
Fungi	CK	3.068 ± 0.093c	0.124 ± 0.024a	556.685 ± 21.119a	582.915 ± 58.951a
	B	3.521 ± 0.313b	0.076 ± 0.030b	593.301 ± 48.953a	591.300 ± 64.239a
	NPK	4.016 ± 0.048a	0.040 ± 0.006b	588.462 ± 31.592a	587.318 ± 30.528a
	NPKB	3.792 ± 0.164ab	0.048 ± 0.008b	629.817 ± 47.811a	615.185 ± 56.105a
	NPKM	3.527 ± 0.217b	0.076 ± 0.027b	615.681 ± 33.391a	604.329 ± 52.598a
	NPKMB	3.898 ± 0.247ab	0.049 ± 0.013b	620.290 ± 46.357a	619.777 ± 54.715a

Different lowercase letters indicate significant differences at *P* < 0.05.

**Figure 2:**
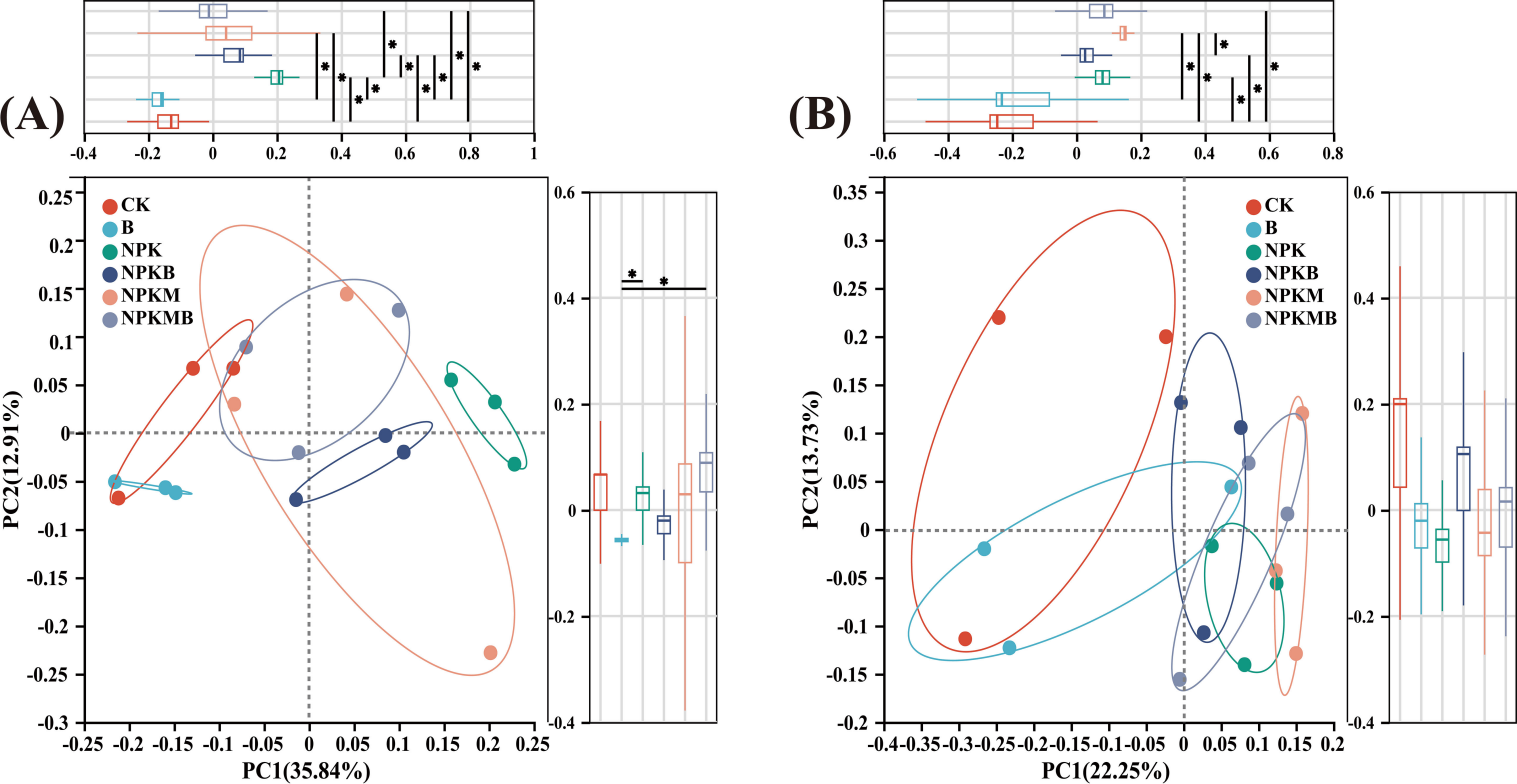
Soil microbial *beta* diversity under contrasting fertilization regimes. Principal coordinate analysis (PCoA) based on Bray-Curtis distances for bacteria **(A)** and fungi **(B)**.

To evaluate the differences in beta-diversity, we performed principal coordinate analysis (PCoA) (**Figure 2**). The first two canonical axes captured 35.84% and 22.25% of variation in bacterial and fungal communities, respectively. Both communities separated significantly along PC1, indicating that long-term contrasting fertilization regimes markedly altered microbial composition in yellow cinnamon soil.

### 3.3 Microbial community composition

**Figure 3:**
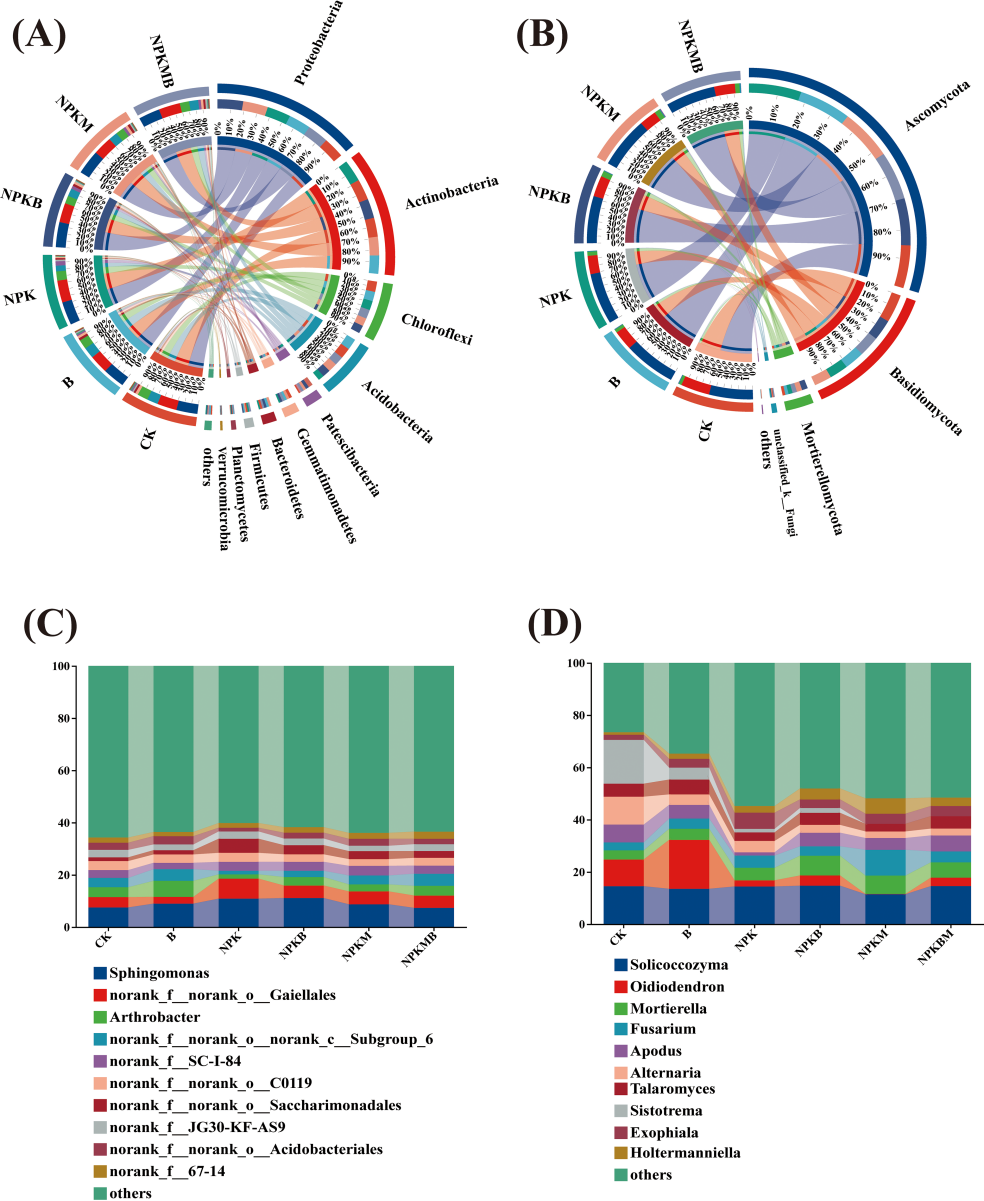
Changes in microbial community composition across long-term fertilization treatments. Relative abundance of bacterial **(A)** and fungal **(B)** phyla, and of dominant bacterial **(C)** and fungal **(D)** genera under each treatment.

High-throughput sequencing revealed diverse bacterial and fungal communities, resolving bacteria into 31 phyla and 714 genera and fungi into 13 phyla and 308 genera. Proteobacteria (29.28-35.68%), Actinobacteria (25.90-31.14%), Chloroflexi (10.90-14.94%), Acidobacteria (7.92-15.54%), Patescibacteria (1.80-6.41%), and Gemmatimonadetes (2.11-3.94%) dominated the bacterial community, collectively exceeding 91% of total abundance (**Figure 3A**). Other phyla exceeding 1% abundance included Bacteroidetes (2.13-3.59%), Firmicutes (1.62-2.56%), and Planctomycetes (0.78-1.27%). Relative to CK, NPK markedly increased Patescibacteria abundance but reduced Acidobacteria, and Gemmatimonadetes also declined under NPK and NPKB (*P* < 0.05). Across all soil samples, the top 10 bacterial genera together comprised less than 40% of total abundance (**Figure 3C**) and belonged to five phyla, namely Proteobacteria, Actinobacteria, Acidobacteria, Chloroflexi and Patescibacteria. Biochar-amended treatments generally showed higher relative abundances of these dominant genera than non-biochar treatments, though the differences were not significant. Notably, g:norank_f:norank_o:Gaiellales peaked under NPK, whereas *Arthrobacter* in the B (biochar only) treatment significantly exceeded that in all other treatments.

Turning to fungi, Ascomycota dominated the community, comprising 57.51% (CK), 68.61% (B), 69.29% (NPK), 64.03% (NPKB), 68.22% (NPKM), and 64.86% (NPKMB) of sequences, followed by Basidiomycota and Mortierellomycota; these three phyla together accounted for over 98% of the fungal community (**Figure 3B**). Fertilization did not markedly alter the relative abundance of these dominant phyla. The most abundant fungal genera across all samples included *Solicoccozyma*, *Oidiodendron*, *Mortierella*, *Fusarium*, *Apodus*, *Alternaria*, *Talaromyces*, *Sistotrema*, *Exophiala*, and *Holtermanniella*, together accounting for 37.13-79.61% of sequences (**Figure 3D**). Relative to B, NPK and NPKM significantly reduced *Oidiodendron* abundance (*P* < 0.05). *Sistotrema* declined notably under every fertilization treatment compared with CK (*P* < 0.05). *Exophiala* was prominently lower in CK, B, and NPKB than in NPK (P < 0.05), while NPKM and NPKMB treatments showed intermediate and non-significant reductions.

**Figure 4:**
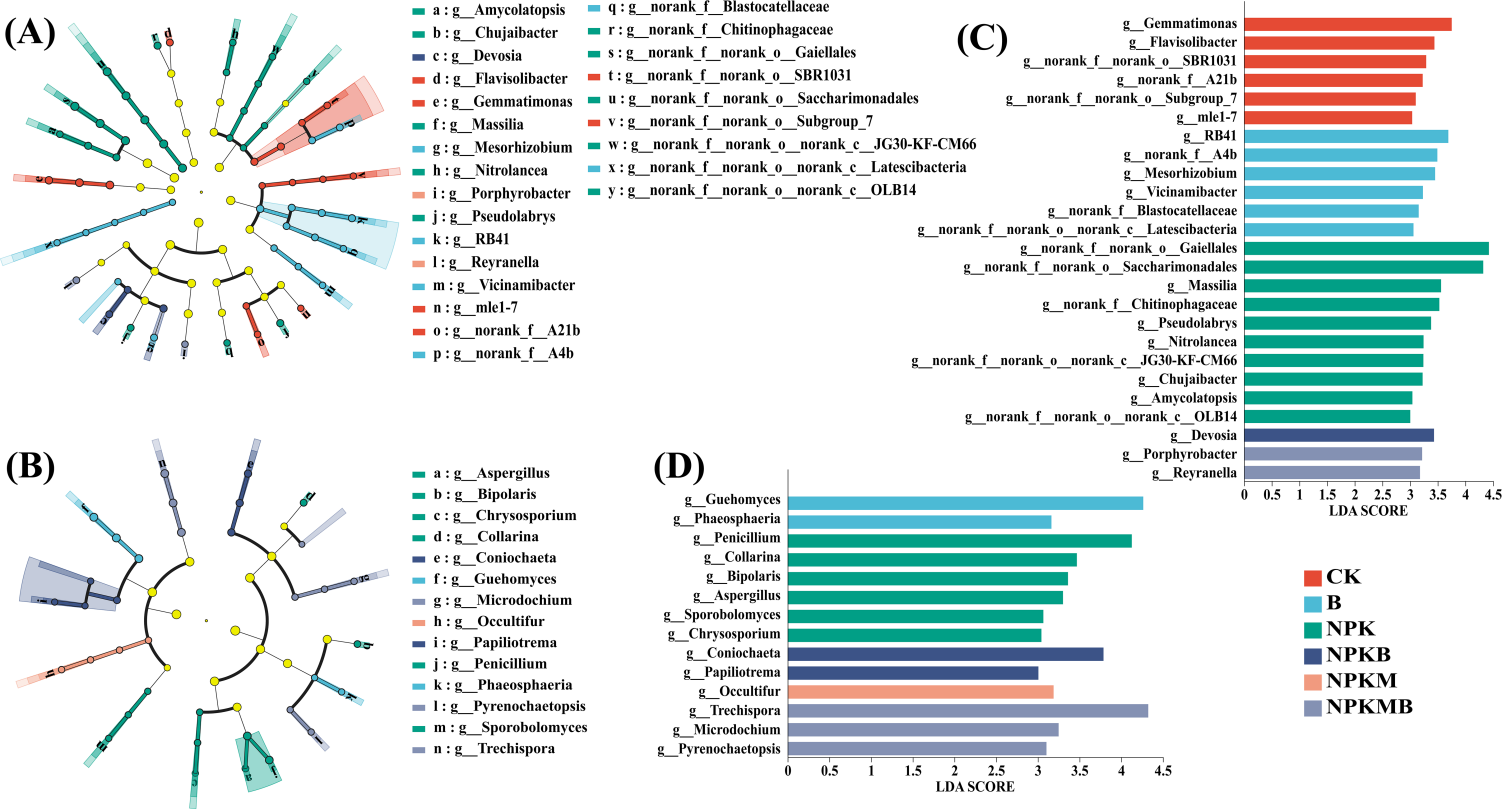
LEfSe cladograms highlighting discriminant taxa (LDA score>3; *P* < 0.05) in bacteria **(A)** and fungi **(B)**. Each ring corresponds to a taxonomic rank from phylum to genus. The LDA scores for bacterial **(C)** and fungal **(D)** biomarkers appear alongside. Colored nodes mark taxa significantly enriched in specific groups and driving group separation; pale yellow nodes indicate taxa lacking significant enrichment or discriminatory power. Only genus-level differences are shown.

We identified discriminant biomarkers between biochar and non-biochar treatments using the Kruskal-Wallis test and linear discriminant analysis (LEfse) (LDA>3). Among bacteria, 63 taxa differed significantly, with 15 biomarkers enriched in CK, 19 in B, 24 in NPK, 3 in NPKB, and 2 in NPKMB. Biochar-associated biomarkers belonged to Acidobacteria and Proteobacteria. In fungi, 36 taxa differed significantly, yielding 5 biomarkers in B, 11 in NPK, 7 in NPKB, 4 in NPKM, and 9 in NPKMB. At the genus level, LEfSe identified *Guehomyces* and *Phaeosphaeria* in B, *Coniochaeta* and *Papiliotrema* in NPKB, and *Trechispora*, *Microdochium*, and *Pyrenochaetopsis* in NPKMB (**Figure 4**).

### 3.4 Contribution of soil microorganisms to soil properties and wheat yield

**Figure 5:**
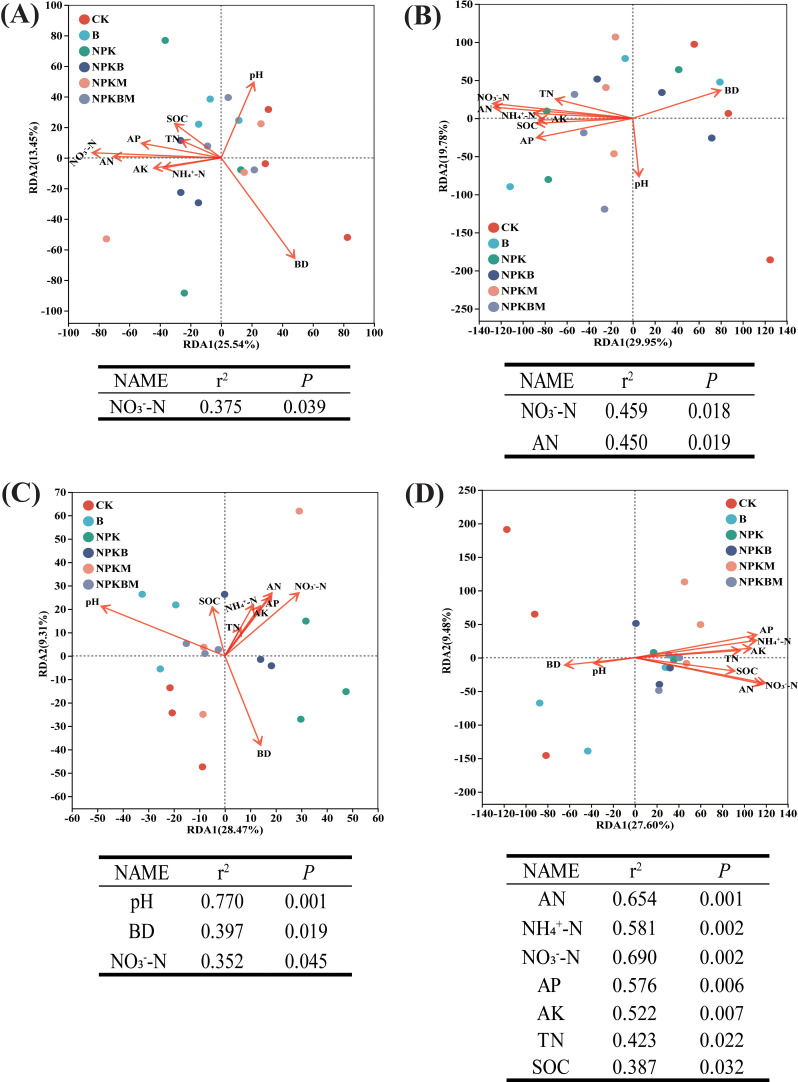
Redundancy analysis (RDA) of bacterial phyla **(A)**, fungal phyla **(B)**, bacterial genera **(C)**, and fungal genera **(D)** in relation to soil physicochemical factors. Statistically significant factors and their explained variation are listed beneath each panel.

Environmental factors strongly shaped the microbial community structure at two taxonomic resolutions, as revealed by RDA (**Figure 5**). At the OTU level, the first two axes captured 40.63% of bacterial variation (27.16% and 13.47%), with NO_3_^--^N exerting the strongest influence (*P* < 0.05), followed by BD, AN, and AP. Fungal communities were even more strongly constrained, with the first two axes accounting for 49.77% (29.64% and 20.13%); both AN and NO_3_^--^N significantly structured this community (*P* < 0.05). Genus-level analysis similarly identified pH, BD and NO_3_^--^N as significant influencers of bacterial assemblages (*P* < 0.05), while AN, NH_4_^+^-N, NO_3_^--^N, AP, AK, TN and SOC significantly affected fungal assemblages (*P* < 0.05). Overall, NO_3_^--^N and AN were principal drivers of both bacterial and fungal communities.

**Figure 6:**
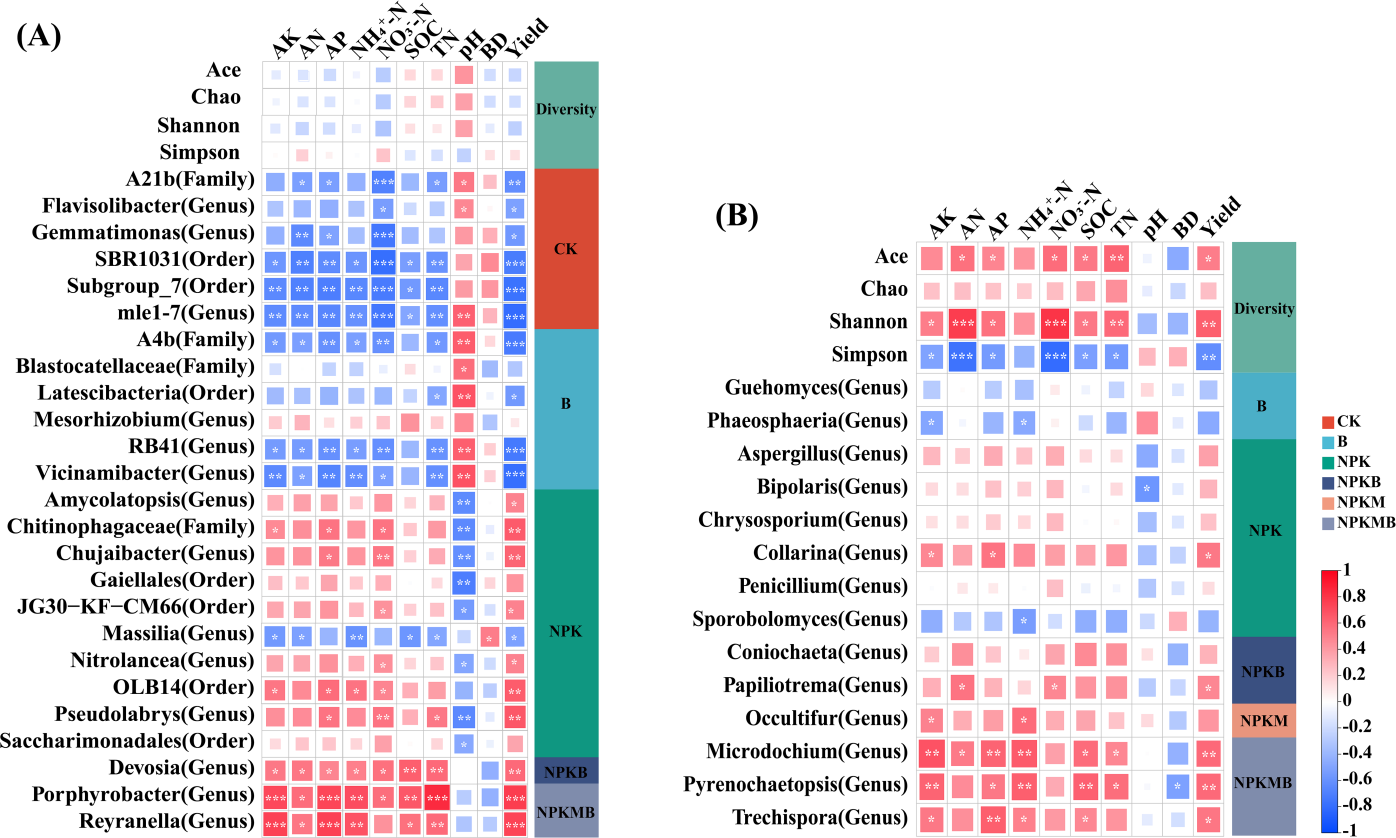
Spearman correlation heat maps of environmental variables, microbial diversity indices, and biomarker taxa. Red indicates positive correlations and blue indicates negative correlations (*P < 0.05; **P < 0.01; ***P < 0.001). A21b (Family): g:norank_f:A21b; A4b (Family): g:norank_f:A4b; Blastocatellaceae (Family): g:norank_f:Blastocatellaceae; Chitinophagaceae (Family): g:norank_f:Chitinophagaceae; Gaiellales (Order): g:norank_f:norank_o:Gaiellales; JG30-KF-CM66 (Order): g:norank_f:norank_o:norank_c:JG30-KF-CM66; Latescibacteria (Order): g:norank_f:norank_o:norank_c:Latescibacteria; OLB14 (Order): g:norank_f:norank_o:norank_c:OLB14; Saccharimonadales (Order): g:norank_f:norank_o:Saccha-rimonadales; SBR1031 (Order): g:norank_f:norank_o:SBR1031; Subgroup_7 (Order): g:norank_f:nor-ank_o:Subgroup_7. **(A)** Correlation heatmap of bacterial biomarkers with soil properties; **(B)** Correlation heatmap of fungal biomarkers with soil properties.

Spearman correlation analysis further linked microbial diversity and biomarkers to soil conditions (**Figure 6**). Fungal ACE and Shannon indices correlated positively with AN, AP, NO_3_^--^N, SOC and TN (*P* < 0.05). In CK and B treatments, bacterial and fungal biomarkers correlated negatively with most environmental factors except pH and BD (*P* < 0.05, *P* < 0.01, or *P* < 0.001), while bacterial biomarkers specifically correlated positively with pH (*P* < 0.05 or *P* < 0.01). Under NPK, bacterial biomarkers (Massilia excepted) correlated positively with soil nutrients and negatively with pH. Biochar-enriched biomarkers in NPKB and NPKMB followed a similar pattern, correlating positively with most factors except pH and BD. Thus, the biomarkers in CK and B trended oppositely to those in NPK, NPKB, and NPKMB across identical environmental factors, whereas pH and BD consistently correlated inversely relative to other soil properties.

## 4 Discussion

### 4.1 Effects of biochar combined fertilization on soil chemical properties

Soil fertility depends on nutrient availability and physical conditions, both of which govern crop productivity (Kooch et al., 2024). Liu et al. (2021) reported that long-term organic fertilizer substitution elevates organic matter and nutrients levels while mitigating soil acidification, thereby promoting crop yield. This finding is corroborated by our results that replacing part of the mineral fertilizer with manure significantly increased SOC (*P* < 0.05; **Table A2**), probably because manure is rich in organic substrates that stimulate microbial mineralization and subsequent nutrient releases (Wu et al., 2022). Correspondingly, under the NPKM treatment, TN, SOC, NH_4_^+^-N, and AK all exceeded levels observed under CK and NPK, with wheat yield rising accordingly (**Table A2**; **Figure 1**). This stems partly from the synergistic effects of combining organic and inorganic inputs, which slows nutrient release and loss, thereby improving utilization efficiency while simultaneously increasing crop yield and quality (Guo et al., 2023). In addition, long-term mineral fertilization lowered soil pH in our study, whereas manure restored pH to near-unfertilized levels, reflecting manure’s capacity to buffer soil acidity where heavy application raises pH in acidic soils yet lowers it in alkaline soils (Guo et al., 2024). Through these improvements in soil properties, organic fertilization directly boosts productivity. For instance, Luo et al. (2018) reported in a meta-analysis that organic amendments raise yields by 27% over mineral fertilizer alone. In our trial on yellow cinnamon soil, manure increased wheat and maize yield by 6.68% and 9.12%, respectively (**Figure 1**).

Biochar addition significantly raised wheat and maize yield by 5.16-85.56% over CK (**Figure 1**), likely due to substantially improved soil quality, including higher SOC and AK (Liu et al., 2020) alongside reduced bulk density (Zhang et al., 2021). Indeed, biochar-amended plots generally outperformed non-biochar plots in nutrient status, with significantly higher SOC and AK. Studies have shown that biochar carries substantial organic carbon (Herath et al., 2013) that gradually mineralizes in soil and becomes available to microorganisms (Roberts et al., 2015). Moreover, Liu et al. (2018) reported that feedstock potassium (K) is largely retained during pyrolysis, and over half of total K in biochar occurs as plant-available K, thereby supplementing soil K reserves for crop uptake. Biochar also buffered soil acidity, raising pH by 0.03-0.15 units over non-biochar treatments (**Table A2**). This effect arises because biochar can exchange with H^+^ and Al³^+^ in soil solution, reducing ion concentrations (Van Zwieten et al., 2010), which is a mechanism previously shown to increase pH in acidic soils (Nielsen et al., 2018). Bulk density is an important indicator of soil physical properties and it also responded favorably to biochar. Lower bulk density improves soil structure, facilitates nutrient release and retention, and alleviates soil compaction. Since biochar has a large surface area, it effectively lowers bulk density (Villagra-Mendoza and Horn, 2018), and our biochar treatments indeed reduced bulk density compared to CK, matching findings by Laird et al. (2010). Overall, manure substitution for mineral fertilizer increased soil pH, nutrient contents, and wheat yield in yellow cinnamon soil, effects that were further amplified by biochar amendment.

### 4.2 Effects of biochar and manure amendments on soil microbial community structure

Soil microbial diversity underpins ecosystem functioning, specifically greater diversity generally confer stronger resistance to environmental interference and enhanced ecosystem stability (Wang et al., 2024b). While long-term exclusive chemical fertilization reduces bacterial diversity, the integration of mineral and organic inputs restores it (van der Bom et al., 2018). Our results align with this pattern, showing that long-term NPK application markedly lowered bacterial richness and diversity, whereas biochar or manure buffered these losses, restoring diversity to near-unfertilized level. The ACE and Chao indexes of NPK treatment were significantly lower than those of other treatments (**Table 2**). This might be because biochar and manure could mitigate the diversity losses typically caused by soil acidification (Sun et al., 2015). Fungal diversity also responded to fertilization (**Table 2**), with NPK showing the highest Shannon index, significantly exceeding levels in B and NPKM. The ACE and Chao indexes under NPK trailed those of other treatments, though not significantly. Notably, environmental factors correlated weakly with bacterial diversity but strongly with fungal metrics, showing significant positive links to ACE and Shannon and negative links to Simpson (**Figure 6**). The divergent responses to soil nutrients reflect fundamental differences in bacterial and fungal life-history strategies (Wang et al., 2024a). Bacteria are usually highly sensitive to available nutrients, so fertilization directly selects dominant taxa by altering soil N, P, and C availability, thereby producing marked shifts in community structure (Fierer et al., 2012). However, diversity itself remains relatively stable because taxon replacement maintains overall richness, which explains the non-significant correlations with soil nutrients (Zhao et al., 2022). By contrast, fungal communities are structurally more stable and less responsive than bacteria to organic substitution (Ren et al., 2021). PCoA at the OTU level further confirmed that fertilization significantly changed bacterial community structure (R²=0.48817, P = 0.001), whereas fungal communities (R²=0.36411, P = 0.031) clustered more tightly across treatments (**Figure 2**). Thus, long-term organic amendments reshaped bacterial communities more profoundly than fungal communities in yellow cinnamon soil.

At the phylum level, fertilization restructured the bacterial community. Relative to CK, NPK significantly reduced Acidobacteria and Gemmatimonadetes, slightly lowered Bacteroidetes, and markedly enriched Patescibacteria (**Figure 3A**). This shift stems from the low soil pH and high inorganic nitrogen that chemical fertilizers create, where acidification reduces Acidobacteria (Li et al., 2020), whereas nitrate-rich conditions favor Patescibacteria (Li et al., 2024). Biochar and manure both offset these negative effects, though in different ways. NPKM strongly influenced Bacteroidetes and Gemmatimonetes, while B treatment significantly restored Acidobacteria and Gemmatimonadetes (**Figure 3A**). This difference arises because manure and biochar supply complex organic carbon that fuels bacteria engaged in nutrient cycling (Wu et al., 2025). Previous research shows that Gaiellales abundance correlates positively with soil NO_3_^-^-N (Chen et al., 2022), explaining why this taxon dominated under NPK (**Figure 3C**). The nitrate rich conditions under exclusive mineral fertilization thus gave this taxon a competitive advantage. *Arthrobacter* is a well-known plant growth-promoting rhizobacterium (PGPR) capable of nitrogen fixation, phosphorus solubilization, phytohormone secretion, and disease suppression. Biochar amendment favors colonization by these beneficial bacteria (Kari et al., 2021), which is a pattern observed in our B treatment. Moreover, combining mineral fertilizer with biochar (NPKB and NPKMB) significantly enriched *Devosia*, *Porphyrobacter* and *Reyranella* (**Figure 4A**; **Table A3**), taxa that correlated positively with most physicochemical factors except pH and BD (**Figure 6A**). All three belong to *Alphaproteobacteria*, a copiotrophic class characterized by rapid growth and wide substrate utilization (Zhang et al., 2016), and are therefore central to nutrient mobilization and plant growth promotion (Lian et al., 2022). Collectively, combining mineral fertilizer with manure and/or biochar not only reshaped bacterial composition but also enriched functional groups such as *Alphaproteobacteria*, thereby strengthening feedback loops between nutrient mobilization and plant growth.

Fertilization also restructured the fungal community at the genus level. *Oidiodendron*, an ericoid mycorrhizal fungus adapted to low-nutrient and low-SOM soils where it assists hosts in acquiring nitrogen (Lin et al., 2020), declined significantly under NPK and NPKM relative to B (P < 0.05; **Figure 3D**). The low-nutrient status under sole biochar application thus provided a suitable ecological niche for this fungus. Conversely, high nutrient availability under NPK and NPKM reduced plant reliance on mycorrhizal symbiosis, inhibiting its growth and colonization (Lehmann et al., 2011). *Sistotrema* also declined significantly under all fertilized treatments relative to CK (P < 0.05; **Figure 3D**). By introducing exogenous nutrients, fertilization reshaped competitive dynamics within the fungal community, allowing fast-growing saprotrophs to dominate while *Sistotrema* declined, probably owing to its weaker competitive capacity under nutrient-rich conditions (Xiang et al., 2020). *Exophiala*, a dark septate endophyte with versatile nitrogen metabolism (Vohník et al., 2005), thrived under NPK because the treatment supplied sufficient nutrients without introducing a large number of organic competitors, creating stable conditions for this taxon (**Figure 3D**). Biochar addition (B and NPKB) may have suppressed *Exophiala* by modifying the soil microenvironment (Lehmann et al., 2011). By contrast, manure application (NPKM and NPKMB) introduced abundant carbon and exogenous microorganisms that raised overall diversity, weakening the dominance of *Exophiala* without prominently reducing its abundance. Similar to their bacterial counterparts, the fungal biomarkers *Trechispora* and *Pyrenochaetopsis* enriched under NPKMB correlated positively with environmental factors except pH and bulk density, and showed significant positive links to AK, AP, NH_4_^+^-N, and SOC (P < 0.05; **Figures 4B**, **6B**). This pattern suggests that combining mineral fertilizer, manure, and biochar established a high-fertility microenvironment with greater carbon input, intensified nutrient cycling, and more complex fungal networks. Specifically, *Trechispora*, a Basidiomycota genus with saprotrophic or facultatively symbiotic habits, correlated positively with SOC because the abundant organic carbon from manure and biochar supplied the energy needed for its proliferation and decomposition activity (Cheng et al., 2023). In contrast, *Pyrenochaetopsis* is a recognized phosphate-solubilizing fungus. Its strong association with AP indicates that even under exogenous P input, this genus mobilizes sparingly soluble phosphorus through organic acid secretion, thereby raising P availability and creating a positive feedback loop with soil AP (Sharma et al., 2013).

In summary, contrasting fertilization regimes reshaped specific microbial groups by altering nutrient availability, microenvironmental conditions, and competitive dynamics. The findings provide empirical evidence linking agricultural management to soil microbial community structure.

## 5 Conclusion

Based on a long-term experiment in yellow cinnamon soil, we evaluated the effects of biochar and manure on soil properties, microbial communities, and wheat yield. Our results demonstrated that these organic amendments significantly raised nutrient levels, restored soil pH, and reduced bulk density, thereby improving overall soil physical and chemical quality. These improvements created a more favorable soil environment, which in turn stimulated microbial biomass and activity. Among all treatments, the combined application of biochar and manure with mineral fertilizer (NPKMB) performed best. This treatment not only improved soil physicochemical properties but also significantly restructured the microbial community, enriching copiotrophic Alphaproteobacteria and saprotrophic/phosphate-solubilizing fungi. These taxa correlated positively with available nutrients and SOC, suggesting their potential roles in nutrient cycling and plant growth promotion. Consequently, the NPKMB treatment established a positive feedback loop whereby improved soil structure enhanced microbial diversity and activity, which in turn increased nutrient mobilization, ultimately raising wheat and maize yield by 85.56% and 67.64%, respectively. This microbial-mediated mechanism is key to the sustainable management of yellow cinnamon soil. In conclusion, combining manure substitution with biochar offers a sustainable strategy for improving soil fertility, beneficial microbial communities, and crop yield in yellow cinnamon soil.

## Data Availability

The data presented in this study are deposited in the National Microbiology Data Center (NMDC) repository, accession number NMDC10020748.
