# Supplementary material for: Effects of biochar and manure amendments on soil microbial communities and crop yield in yellow cinnamon soil
Source: Front Plant Sci. 2026 Jul 7;17:1884267. doi: 10.3389/fpls.2026.1884267 (PMC13385055; doi:10.3389/fpls.2026.1884267)
Supplement: Supplementary file 1 [file DataSheet1.docx]

**Supplementary Materials**

**Text 1**

Peanut shell biochar, provided by Sanli New Energy Company (Henan Province, China), was used in this experiment. Production procedures followed Pan et al. (2011): feedstock was pyrolyzed in a vertical kiln at a about 500 °C, yielding roughly 30% biochar, 250 kg bioliquid (wood vinegar and pyrolysis oil), and 800 m^3^ syngas per ton of feedstock. Before application, biochar was ground to pass a 2 mm sieve and homogenized. The properties were determined following Bao (2000): total C 647 g·kg^−1^, total N 15.22 g·kg^−1^, pH (H_2_O) 9.16, specific surface area 12.13 m^2^·g^−1^, ash content 2.41%, and CEC 148 cmol·kg^−1^.

Commercial chicken manure served as the organic amendment, containing 12 g·kg^−1^ N, 283 g·kg^−1^ organic C, 642 g·kg^−1^ available K, and having a pH of 8.16.

**Text 2**

Soil properties analysis followed Bao [57]. pH was measured using a glass electrode (DZS-707; Zhejiang Nade Scientific Instrument Co., Ltd., Shanghai, China) in a 1:2.5 (w:w) soil-to-water suspension. Soil moisture content was measured gravimetrically after drying at 105 °C. Nitrate (NO_3_^-^-N) and ammonium (NH_4_^+^-N) were analyzed by continuous flow analyzer (SKALAR SAN++，Netherland). SOC was determined by K_2_Cr_2_O_7_-FeSO_4_ oxidation, and TN by Kjeldahl digestion (KDN-102C, Shanghai, China). AN, AP, and AK were measured by alkaline diffusion, Olsen, and ammonium acetate extraction methods, respectively.


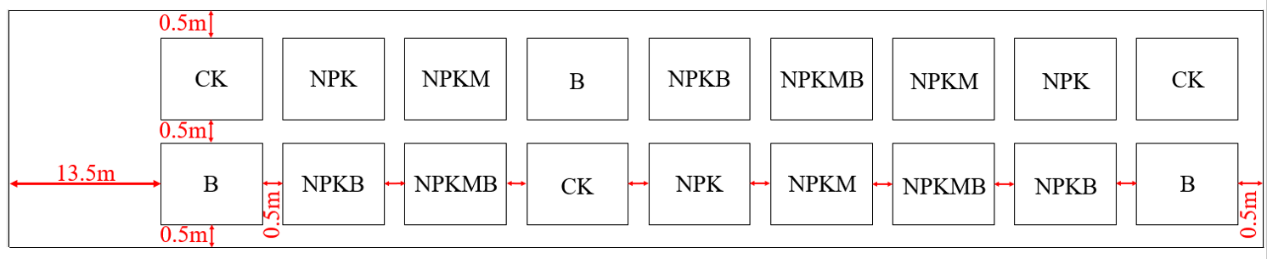


1. Layout of the study plots.


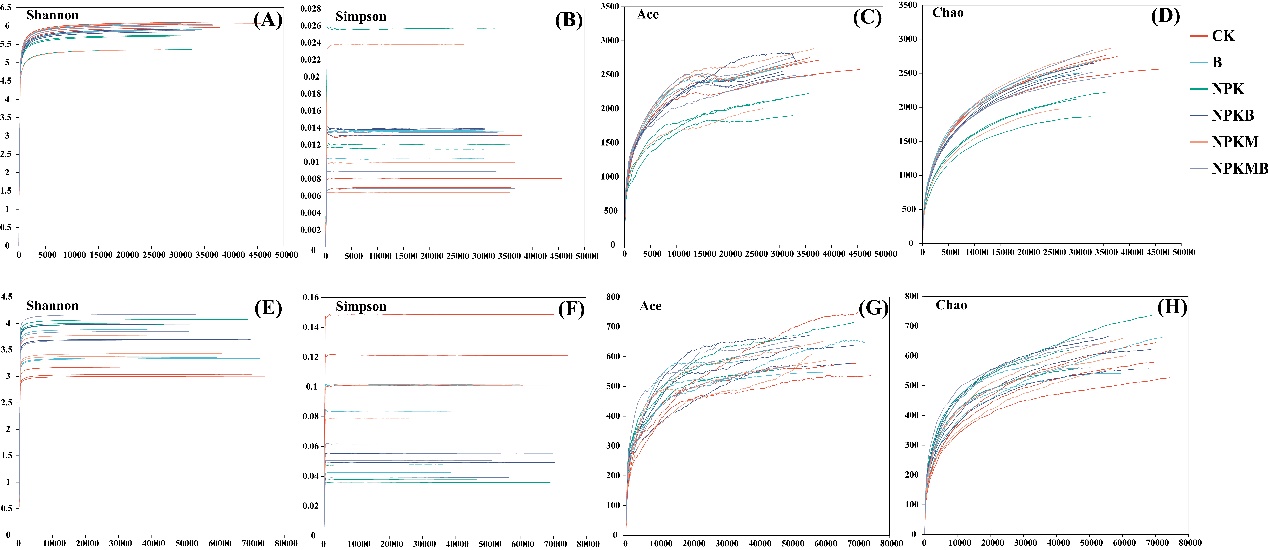


1. Rarefaction curves showing the relationships between bacterial α-diversity and sequencing depth.
2. **Annual nutrient inputs（kg ha^-1^ year^-1^）**

| Treatment | Winter wheat | | | | |  | Summer maize | | |
| --- | --- | --- | --- | --- | --- | --- | --- | --- | --- |
|  | N | P_2_O_5_ | K_2_O | M | B |  | N | P_2_O_5_ | K_2_O |
| CK | 0 | 0 | 0 | 0 | 0 |  | 0 | 0 | 0 |
| B | 0 | 0 | 0 | 0 | 4 500 |  | 0 | 0 | 0 |
| NPK | 180 | 90 | 75 | 0 | 0 |  | 210 | 75 | 90 |
| NPKB | 180 | 90 | 75 | 0 | 4 500 |  | 210 | 75 | 90 |
| NPKM | 72 | 90 | 75 | 9 000 | 0 |  | 210 | 75 | 90 |
| NPKMB | 72 | 90 | 75 | 9 000 | 4 500 |  | 210 | 75 | 90 |

Note：The total N application was identical across wheat-season treatments, with a basal-to-topdressing ratio of 1:1 for inorganic N. Basal fertilizer was broadcast before wheat sowing, with topdressing applied in furrows at the jointing stage. P, K, manure and biochar were applied entirely as basal fertilizer before wheat sowing, followed by rotary tillage and land preparation. All fertilizers were applied annually during the experimental period. During the maize season, only chemical fertilizers were used; P and K were applied once at the five-leaf stage, while N was split equally (5:5) between the five-leaf and tasseling stages. Fertilizer sources were urea (46% N), superphosphate (12% P_2_O_5_), and potassium chloride (50% K_2_O).

1. **Soil properties under different fertilization treatments.**

| Treatment | pH | BD  ( g·cm^-3^) | TN  (g·kg^-1^) | SOC  ( g·kg^-1^) | NH_4_^+^-N  (mg·kg^-1^) | NO_3_^-^ -N  (mg·kg^-1^) | | AN  (mg·kg^-1^) | AP  ( mg·kg^-1^) | AK  ( mg·kg^-1^) |
| --- | --- | --- | --- | --- | --- | --- | --- | --- | --- | --- |
| CK | 5.40±0.00ab | 1.49±0.05a | 1.17±0.03d | 10.42±0.24e | 5.13±0.04d | 16.00±0.31d | | 52.67±1.15c | 15.90±1.28d | 120.33±4.04f |
| B | 5.50±0.17a | 1.38±0.00bc | 1.27±0.03cd | 14.22±0.46c | 5.95±0.21c | 19.77±0.24c | 79.50±4.50b | | 19.00±0.56d | 136.67± 6.35e |
| NPK | 5.15±0.05c | 1.44±0.03b | 1.35±0.07c | 12.83±0.43d | 6.98±0.11b | 20.74±0.46a | | 81.00±3.00b | 24.35±2.55bc | 162.67±4.16d |
| NPKB | 5.30±0.10bc | 1.42±0.03b | 1.52±0.08b | 15.67±0.32b | 7.34±0.13b | 20.26±0.58abc | | 90.00±12.29ab | 23.67±1.03c | 172.67±4.04c |
| NPKM | 5.35±0.05ab | 1.41±0.04b | 1.51±0.12b | 15.67±1.12b | 9.95±0.15a | 19.89±0.26bc | | 82.50±1.50b | 27.73±1.50b | 200.00±7.21b |
| NPKMB | 5.38±0.03ab | 1.35±0.02c | 1.72±0.12a | 20.22±0.81a | 10.18±0.40a | 20.55±0.29ab | | 96.00±0.00a | 33.23±3.16a | 237.33±2.08a |

Note: Values are means (n = 3) ± SD (standard deviation). Different lowercase letters within a column indicate significant differences at *P* < 0.05. BD, bulk density; TN, total nitrogen; SOC, soil organic carbon; NH_4_^+^-N, ammonium nitrogen; NO_3_^-^-N, nitrate nitrogen; AN, available nitrogen; AP, available phosphorus; AK, available potassium.

1. **Bacterial biomarkers with LDA scores > 3.**

| **Treatment** | **Phylum** | **Class** | **Order** | **Family** | **Genus** |
| --- | --- | --- | --- | --- | --- |
| B | p__Acidobacteria | c__Blastocatellia_Subgroup_4 | | |  |
| B | p__Acidobacteria | c__Blastocatellia_Subgroup_4 | o__Pyrinomonadales | f__Pyrinomonadaceae | |
| B | p__Acidobacteria | c__Blastocatellia_Subgroup_4 | o__Pyrinomonadales | |  |
| B | p__Acidobacteria | c__Blastocatellia_Subgroup_4 | o__Pyrinomonadales | f__Pyrinomonadaceae | g__RB41 |
| B | p__Acidobacteria | c__Blastocatellia_Subgroup_4 | o__Blastocatellales | f__Blastocatellaceae | |
| B | p__Acidobacteria | c__Blastocatellia_Subgroup_4 | o__Blastocatellales | |  |
| B | p__Acidobacteria | c__Subgroup_6 | o__unclassified | |  |
| B | p__Acidobacteria | c__Subgroup_6 | o__unclassified | f__unclassified_o__unclassified | g__Vicinamibacter |
| B | p__Acidobacteria | c__Subgroup_6 | o__unclassified | f__unclassified_o__unclassified | |
| B | p__Acidobacteria | c__Blastocatellia_Subgroup_4 | o__Blastocatellales | f__Blastocatellaceae | g__norank_f__Blastocatellaceae |
| B | p__Chloroflexi | c__Anaerolineae | o__SBR1031 | f__A4b |  |
| B | p__Chloroflexi | c__Anaerolineae | o__SBR1031 | f__A4b | g__norank_f__A4b |
| B | p__Latescibacteria | c__Latescibacteria | o__norank_c__Latescibacteria | f__norank_o__norank_c__Latescibacteria | |
| B | p__Latescibacteria | c__Latescibacteria | |  |  |
| B | p__Latescibacteria | c__Latescibacteria | o__norank_c__Latescibacteria | f__norank_o__norank_c__Latescibacteria | g__norank_f__norank_o__norank_c__Latescibacteria |
| B | p__Latescibacteria | c__Latescibacteria | o__norank_c__Latescibacteria | | |
| B | p__Latescibacteria |  |  |  |  |
| B | p__Proteobacteria | c__Alphaproteobacteria | o__Rhizobiales | f__Rhizobiaceae | g__Mesorhizobium |
| B | p__Proteobacteria | c__Alphaproteobacteria | o__Rhizobiales | f__Rhizobiales_Incertae_Sedis | |
| CK | p__Acidobacteria | c__Holophagae | o__Subgroup_7 | |  |
| CK | p__Acidobacteria | c__Holophagae | o__Subgroup_7 | f__norank_o__Subgroup_7 | g__norank_f__norank_o__Subgroup_7 |
| CK | p__Acidobacteria | c__Holophagae | |  |  |
| CK | p__Acidobacteria | c__Holophagae | o__Subgroup_7 | f__norank_o__Subgroup_7 | |
| CK | p__Bacteroidetes | c__Bacteroidia | o__Chitinophagales | f__Chitinophagaceae | g__Flavisolibacter |
| CK | p__Chloroflexi | c__Anaerolineae | |  |  |
| CK | p__Chloroflexi | c__Anaerolineae | o__SBR1031 | |  |
| CK | p__Chloroflexi | c__Anaerolineae | o__SBR1031 | f__norank_o__SBR1031 | |
| CK | p__Chloroflexi | c__Anaerolineae | o__SBR1031 | f__norank_o__SBR1031 | g__norank_f__norank_o__SBR1031 |
| CK | p__Gemmatimonadetes | c__Gemmatimonadetes | o__Gemmatimonadales | | |
| CK | p__Gemmatimonadetes | c__Gemmatimonadetes | o__Gemmatimonadales | f__Gemmatimonadaceae | |
| CK | p__Gemmatimonadetes | c__Gemmatimonadetes | o__Gemmatimonadales | f__Gemmatimonadaceae | g__Gemmatimonas |
| CK | p__Proteobacteria | c__Gammaproteobacteria | o__Betaproteobacteriales | f__A21b | g__norank_f__A21b |
| CK | p__Proteobacteria | c__Gammaproteobacteria | o__Betaproteobacteriales | f__A21b |  |
| CK | p__Proteobacteria | c__Gammaproteobacteria | o__Betaproteobacteriales | f__Nitrosomonadaceae | g__mle1-7 |
| NPK | p__Actinobacteria | c__Actinobacteria | o__Gaiellales | f__norank_o__Gaiellales | |
| NPK | p__Actinobacteria | c__Actinobacteria | o__Gaiellales | f__norank_o__Gaiellales | g__norank_f__norank_o__Gaiellales |
| NPK | p__Actinobacteria | c__Actinobacteria | o__Gaiellales | |  |
| NPK | p__Actinobacteria | c__Actinobacteria | o__Pseudonocardiales | f__Pseudonocardiaceae | |
| NPK | p__Actinobacteria | c__Actinobacteria | o__Pseudonocardiales | | |
| NPK | p__Actinobacteria | c__Actinobacteria | o__Pseudonocardiales | f__Pseudonocardiaceae | g__Amycolatopsis |
| NPK | p__Bacteroidetes | c__Bacteroidia | o__Chitinophagales | f__Chitinophagaceae | g__norank_f__Chitinophagaceae |
| NPK | p__Chloroflexi | c__JG30-KF-CM66 | o__norank_c__JG30-KF-CM66 | f__norank_o__norank_c__JG30-KF-CM66 | |
| NPK | p__Chloroflexi | c__JG30-KF-CM66 | |  |  |
| NPK | p__Chloroflexi | c__Chloroflexia | o__Thermomicrobiales | f__Thermomicrobiaceae | g__Nitrolancea |
| NPK | p__Chloroflexi | c__JG30-KF-CM66 | o__norank_c__JG30-KF-CM66 | f__norank_o__norank_c__JG30-KF-CM66 | g__norank_f__norank_o__norank_c__JG30-KF-CM66 |
| NPK | p__Chloroflexi | c__JG30-KF-CM66 | o__norank_c__JG30-KF-CM66 | | |
| NPK | p__Chloroflexi | c__Chloroflexia | o__Thermomicrobiales | f__Thermomicrobiaceae | |
| NPK | p__Chloroflexi | c__OLB14 | o__norank_c__OLB14 | | |
| NPK | p__Chloroflexi | c__OLB14 | |  |  |
| NPK | p__Chloroflexi | c__OLB14 | o__norank_c__OLB14 | f__norank_o__norank_c__OLB14 | g__norank_f__norank_o__norank_c__OLB14 |
| NPK | p__Patescibacteria | c__Saccharimonadia | |  |  |
| NPK | p__Patescibacteria |  |  |  |  |
| NPK | p__Patescibacteria | c__Saccharimonadia | o__Saccharimonadales | | |
| NPK | p__Patescibacteria | c__Saccharimonadia | o__Saccharimonadales | f__norank_o__Saccharimonadales | g__norank_f__norank_o__Saccharimonadales |
| NPK | p__Patescibacteria | c__Saccharimonadia | o__Saccharimonadales | f__norank_o__Saccharimonadales | |
| NPK | p__Proteobacteria | c__Gammaproteobacteria | o__Betaproteobacteriales | f__Burkholderiaceae | g__Massilia |
| NPK | p__Proteobacteria | c__Alphaproteobacteria | o__Rhizobiales | f__Xanthobacteraceae | g__Pseudolabrys |
| NPK | p__Proteobacteria | c__Gammaproteobacteria | o__Xanthomonadales | f__Rhodanobacteraceae | g__Chujaibacter |
| NPKB | p__Proteobacteria | c__Alphaproteobacteria | o__Rhizobiales | f__Rhizobiaceae | |
| NPKB | p__Proteobacteria | c__Alphaproteobacteria | o__Rhizobiales | f__Devosiaceae | |
| NPKB | p__Proteobacteria | c__Alphaproteobacteria | o__Rhizobiales | f__Devosiaceae | g__Devosia |
| NPKBM | p__Proteobacteria | c__Alphaproteobacteria | o__Sphingomonadales | f__Sphingomonadaceae | g__Porphyrobacter |
| NPKBM | p__Proteobacteria | c__Alphaproteobacteria | o__Reyranellales | f__Reyranellaceae | g__Reyranella |

1. **Fungal biomarkers with LDA scores > 3.**

| **Treatment** | **Phylum** | **Class** | **Order** | **Family** | **Genus** |
| --- | --- | --- | --- | --- | --- |
| B | p__Basidiomycota | c__Tremellomycetes | o__Cystofilobasidiales | f__Cystofilobasidiaceae | |
| B | p__Basidiomycota | c__Tremellomycetes | o__Cystofilobasidiales | | |
| B | p__Basidiomycota | c__Tremellomycetes | o__Cystofilobasidiales | f__Cystofilobasidiaceae | g__Guehomyces |
| B | p__Ascomycota | c__Dothideomycetes | o__Pleosporales | f__Phaeosphaeriaceae | |
| B | p__Ascomycota | c__Dothideomycetes | o__Pleosporales | f__Phaeosphaeriaceae | g__Phaeosphaeria |
| NPK | p__Ascomycota | c__Eurotiomycetes | o__Eurotiales | f__Aspergillaceae | |
| NPK | p__Ascomycota | c__Eurotiomycetes | o__Eurotiales | f__Aspergillaceae | g__Penicillium |
| NPK | p__Ascomycota | c__Sordariomycetes | o__Hypocreales | f__Clavicipitaceae | g__Collarina |
| NPK | p__Ascomycota | c__Dothideomycetes | o__Pleosporales | f__Pleosporaceae | g__Bipolaris |
| NPK | p__Ascomycota | c__Eurotiomycetes | o__Eurotiales | f__Aspergillaceae | g__Aspergillus |
| NPK | p__Ascomycota | c__Eurotiomycetes | o__Onygenales | |  |
| NPK | p__Ascomycota | c__Eurotiomycetes | o__Onygenales | f__Onygenales_fam_Incertae_sedis | |
| NPK | p__Basidiomycota | c__Microbotryomycetes | o__Sporidiobolales | |  |
| NPK | p__Basidiomycota | c__Microbotryomycetes | o__Sporidiobolales | f__Sporidiobolaceae | g__Sporobolomyces |
| NPK | p__Basidiomycota | c__Microbotryomycetes | o__Sporidiobolales | f__Sporidiobolaceae | |
| NPK | p__Ascomycota | c__Eurotiomycetes | o__Onygenales | f__Onygenales_fam_Incertae_sedis | g__Chrysosporium |
| NPKB | p__Ascomycota | c__Sordariomycetes | o__Coniochaetales | |  |
| NPKB | p__Ascomycota | c__Sordariomycetes | o__Coniochaetales | f__Coniochaetaceae | |
| NPKB | p__Ascomycota | c__Sordariomycetes | o__Coniochaetales | f__Coniochaetaceae | g__Coniochaeta |
| NPKB | p__Basidiomycota | c__Tremellomycetes | o__Tremellales | |  |
| NPKB | p__Basidiomycota | c__Tremellomycetes | o__Tremellales | f__Bulleribasidiaceae | |
| NPKB | p__Basidiomycota | c__Tremellomycetes | o__Tremellales | f__Rhynchogastremataceae | |
| NPKB | p__Basidiomycota | c__Tremellomycetes | o__Tremellales | f__Rhynchogastremataceae | g__Papiliotrema |
| NPKM | p__Basidiomycota | c__Cystobasidiomycetes | o__Cystobasidiales | |  |
| NPKM | p__Basidiomycota | c__Cystobasidiomycetes | o__Cystobasidiales | f__Cystobasidiaceae | g__Occultifur |
| NPKM | p__Basidiomycota | c__Cystobasidiomycetes | o__Cystobasidiales | f__Cystobasidiaceae | |
| NPKM | p__Basidiomycota | c__Cystobasidiomycetes | | |  |
| NPKBM | p__Basidiomycota | c__Agaricomycetes | o__Trechisporales | f__Hydnodontaceae | g__Trechispora |
| NPKBM | p__Basidiomycota | c__Agaricomycetes | o__Trechisporales | |  |
| NPKBM | p__Basidiomycota | c__Agaricomycetes | o__Trechisporales | f__Hydnodontaceae | |
| NPKBM | p__Ascomycota | c__Sordariomycetes | o__Xylariales | f__Microdochiaceae | g__Microdochium |
| NPKBM | p__Ascomycota | c__Sordariomycetes | o__Xylariales | |  |
| NPKBM | p__Ascomycota | c__Sordariomycetes | o__Xylariales | f__Microdochiaceae | |
| NPKBM | p__Ascomycota | c__Sordariomycetes | o__Hypocreales | f__Cordycipitaceae | |
| NPKBM | p__Ascomycota | c__Dothideomycetes | o__Pleosporales | f__Cucurbitariaceae | |
| NPKBM | p__Ascomycota | c__Dothideomycetes | o__Pleosporales | f__Cucurbitariaceae | g__Pyrenochaetopsis |

**References**

Pan, G., Lin, Z., Li, L., Zhang, A., Zheng, J., and Zhang, X. (2011). Perspective on biomass carbon industrialization of organic waste from agriculture and rural areas in China. *J. Agric. Sci. Technol.* 13, 75–82.

Bao, S. D. (2000). *Soil Agrochemical Analysis*. Beijing: China Agriculture Press, 25–106.
